# Effect of eggshell nanoparticles and fluoride varnish, with and without diode laser, on dentinal tubule occlusion (An in vitro study)

**DOI:** 10.1186/s12903-026-07800-1

**Published:** 2026-02-20

**Authors:** Mona El Sayed, Mona E. Eliwa, Howaida Fakhry Fouad, Reem Zeid

**Affiliations:** 1 Faculty of Dentistry, Ahram Canadian University, Giza, Egypt; 2 Faculty of Dentistry, Galala University, Suez, Egypt

**Keywords:** Dentine hypersensitivity, Nano eggshell, Fluoride varnish, Diode laser, Dentinal tubule occlusion

## Abstract

**Background:**

Dentine hypersensitivity is a common clinical condition, affecting between 4% and 69% of the adult population. Management strategies are based on occluding patent dentinal tubules to reduce dentinal fluid movement. This in vitro study aimed to evaluate the effect of nano eggshell and fluoride varnish, alone and combined with diode laser, on dentinal tubule occlusion using scanning electron microscopy (SEM) and energy dispersive X-ray spectroscopy (EDX).

**Methods:**

Forty sound human maxillary premolars were sectioned to expose mid-coronal dentine and etched with 37% phosphoric acid to simulate hypersensitive dentine. Samples were randomly allocated into five groups (*n* = 8): Group 1 (nano eggshell), Group 2 (fluoride varnish), Group 3 (nano eggshell + diode laser), Group 4 (fluoride varnish + diode laser), and Group 5 (control). Nano eggshell and fluoride varnish were applied daily for seven consecutive days. Groups 3 and 4 received 940 nm diode laser irradiation after completing the 7-day treatment protocol. Group 5 received no treatment. All samples were analyzed using SEM and EDX to assess dentinal tubule occlusion and calcium to phosphorus (Ca/P) ratio. Data were statistically analyzed using one-way ANOVA and Tukey’s post hoc test with significance set at *P* ≤ 0.05.

**Results:**

All treatment groups exhibited significantly lower percentages of patent dentinal tubules and higher Ca/P ratios than the control (*P* < 0.0001). Group 3 (nano eggshell + laser) exhibited the lowest percentage of patent tubules (0.65 ± 0.14%), followed by Group 1 (nano eggshell, 2.33 ± 0.21%), Group 4 (fluoride + laser, 3.84 ± 0.67%), Group 2 (fluoride, 6.73 ± 0.55%), while Group 5 (control) showed the highest percentage (12.95 ± 1.39%). The Ca/P ratio was highest in Group 3 (1.755 ± 0.1226), followed by Groups 1, 4, and 2, respectively, with the control group showing the lowest ratio (0.603 ± 0.09096).

**Conclusions:**

All tested treatments significantly reduced the percentage of patent dentinal tubules and increased Ca/P ratios compared to the control group. The combination of nano eggshell with diode laser demonstrated the highest efficacy. Nano eggshell-based treatments outperformed fluoride varnish, presenting a promising, biocompatible, and cost-effective approach for dentine hypersensitivity management.

## Background

Dentine hypersensitivity is a common clinical condition with high prevalence, ranging from 4% to 69% in the adult population, with peak incidence between the ages of 20–40 years [1]. This condition is characterized by short, sharp pain triggered by tactile, thermal, osmotic, evaporative, or electrical stimuli that is not attributable to any other dental pathology [2]. Consequently, accurate diagnosis requires systematic exclusion of other potential dental pain sources or structural defects [2].

The development of dentine hypersensitivity is associated primarily with clinical factors such as dentinal tubule exposure, gingival recession, and tooth structure loss, including enamel and cementum [3]. Among various proposed theories, the hydrodynamic theory is the most accepted explanation, suggesting that abrupt fluid movement within dentinal tubules leads to stimulation of pulpal nerve fibres, thereby eliciting a sharp, localized pain response [2].

The prevalence of this condition, along with its tendency for recurrence, emphasizes the necessity for appropriate management strategies [4]. Management of dentine hypersensitivity is based on the principle that occluding or narrowing open dentinal tubules reduces dentine permeability and decreases fluid movement within tubules, thereby diminishing pain sensation [5].

Desensitizing agents are commonly employed for the management of dentine hypersensitivity, incorporating active ingredients such as sodium fluoride, sodium monofluorophosphate, amorphous calcium phosphate, and nanohydroxyapatite [6]. The efficacy of fluoride-containing compounds in reducing dentine hypersensitivity is well documented, as sodium fluoride application on sensitive teeth promotes calcium fluoride crystals formation, which block exposed dentinal tubules and reduce dentine permeability in accordance with hydrodynamic theory [7].

In recent years, interest in bioactive materials that can reduce dentinal fluid movement by blocking dentinal tubules has increased [8]. The remineralization potential of eggshells has been investigated in several studies, as they have a rich content of bioavailable calcium in the form of carbonate and oxide compounds, comprising approximately 94% calcium carbonate, 1% calcium phosphate, 1% magnesium carbonate, and 4% organic components. Additionally, they represent a cost-effective, sustainable, and renewable source of nanohydroxyapatite [8].

Laser therapy has been employed as an effective non-invasive therapeutic approach for treating dentine hypersensitivity since its introduction in the mid-1980s [9]. Although the exact mechanism remains incompletely understood, current evidence suggests that lasers reduce hypersensitivity by thermally inducing melting and recrystallization of the exposed dentine surface, thereby sealing the dentinal tubules [10].

Several types of lasers have been evaluated for dentine hypersensitivity treatment, including erbium-doped yttrium-aluminum garnet (Er: YAG), neodymium-doped yttrium-aluminum garnet (Nd: YAG), CO₂, and diode lasers. Among these, diode lasers with different wavelengths have shown significant effectiveness, frequently demonstrating outcomes comparable to or exceeding those of conventional treatments [6]. The 940 nm diode laser offers particular clinical advantages, including cost-effectiveness, portability, and versatility in dental procedures [11].

Combined laser therapy and desensitizing agent treatment has been shown to enhance therapeutic efficacy and has demonstrated clinical effectiveness in reducing dentine hypersensitivity [12]. This study aimed to evaluate the effect of nano eggshell and fluoride varnish, alone and in combination with diode laser, on dentinal tubule occlusion using scanning electron microscopy (SEM) and energy dispersive X-ray spectroscopy (EDX). The null hypothesis was that nano eggshell, fluoride varnish, or their combinations with diode laser would have no significant effect on dentinal tubule occlusion or calcium to phosphorus (Ca/P) ratio of dentine.

## Methods

This study was approved by the Research Ethics Committee of the Faculty of Dentistry, Ahram Canadian University, Giza, Egypt (IRB00012891#152). Sample size calculation was performed using G*Power software (version 3.1.9.4) based on a previous study [13]. A minimum sample size of 8 samples per group was required when the mean and standard deviation of Group I was 78.92 ± 6.05, whereas for Group II it was 89.42 ± 6.99, with an effect size of 1.61, assuming 80% power and type I error probability of 0.05.

### Preparation and characterization of eggshell nanoparticles

Eggshell nanoparticles were prepared by Nano Gate Company, Egypt, following the calcination method described by the World Intellectual Property Organization (WO/2004/105912: Method of Producing Eggshell Powder). Twelve chicken eggs were thoroughly cleaned and boiled at 100 °C for 10 min. The eggshells were crushed using a sterile mortar and pestle, and then calcined in a muffle furnace (Neycraft, JFF 2000, USA) at 1200 °C to ensure sterility [14].

The resulting calcined material was milled using a ball milling machine (PM 400, Retsch, Germany) at a speed of 350 rpm for 10 h with 3 min rest intervals to obtain nanoscale powder. The final product, with a particle size of 35–65 nm, was characterized by the manufacturer via X-ray diffraction analysis (X’Pert PRO Powder Diffractometer, Malvern Panalytical, Netherlands), confirming crystalline structure and nanoscale dimensions of the particles.

### Sample collection and Preparation

Forty sound human maxillary premolars, extracted for orthodontic purposes, were utilized. Teeth were ultrasonically scaled and thoroughly rinsed to remove residual soft tissue, blood, and debris. Each tooth was examined under 3.5× magnification, and those exhibiting caries, cracks, stains, or other structural defects were excluded. The eligible teeth were maintained in 0.1% thymol solution at 4 °C in sealed containers for a maximum period of one month to inhibit bacterial growth and preserve tissue hydration [15].

For sample preparation, the coronal portion of each tooth was measured, and half of it was sectioned using a diamond-coated disc (Isomet 4000, Buehler, IL, USA) under water cooling to expose the mid-coronal dentine [16]. Each sample was mounted in a block of self-cured acrylic resin (Acrostone, Anglo Egyptian Company, Egypt). The occlusal surface was then ground under water cooling using 600–grit silicon carbide paper for 1 min (Dongguan Golden Sun Abrasives Co., Ltd., Dongguan, China), followed by thorough water rinsing to produce uniformly smooth surfaces [16]. To standardize the working area, dentine surfaces were coated with a double layer of acid-resistant nail varnish, exposing a standardized 3 × 4 mm window. Each resin block was then assigned a random identification number.

### Simulation of hypersensitive dentine

To create the hypersensitivity model, dentine samples were etched with 37% phosphoric acid (Meta Biomed, Korea) for 15 s to effectively remove the smear plugs and expose dentinal tubules [17]. Following etching, samples were rinsed with distilled water for 60 s and gently air-dried for an additional 60 s according to the manufacturer’s instructions.

### Experimental grouping and treatment protocol

The samples were randomly allocated to five groups (*n* = 8 each) as follows:

- Group 1 (Nano eggshell): A slurry of nano eggshell was prepared by mixing 1.8 g of nano eggshell powder with 0.3 mL of distilled water [18]. The mixture was applied to the dentine surface with a microbrush, left for 7 min, followed by rinsing with distilled water and gentle air drying. The 7-minute dwell time was selected to ensure adequate and standardized contact between the nano eggshell paste and the exposed dentinal surface, allowing sufficient time for ion release, particle interaction, and initial mineral deposition under static in vitro conditions. This application duration is consistent with previously published in vitro studies evaluating nanoparticle-based desensitizing agents, in which the materials were left undisturbed on the dentine surface for 7 min [18, 19].
- Samples were subsequently immersed in artificial saliva solution that was composed of NaHCO₃ (3.27 mM), Na₃PO₄ (3.90 mM), KCl (17.98 mM), MgCl₂ (0.08 mM), NaCl (4.29 mM), H₂SO₄ (0.50 mM), CaCl₂ (1.10 mM), and distilled water, with pH adjusted to 7.2 [11].
- This procedure was repeated daily for seven consecutive days, with the artificial saliva solution replenished every 24 h following the application of the nano eggshell. The 7-day treatment regimen was selected based on established protocols demonstrating effective dentinal tubule occlusion with repeated daily applications [19, 20]. This duration allows for progressive accumulation of desensitizing materials and formation of stable deposits through multiple precipitation cycles, while representing a clinically practical timeframe that balances treatment efficacy with patient compliance.
- Group 2 (Fluoride): Fluoride varnish (Bifluorid 10, Voco GmbH, Cuxhaven, Germany) was applied to the dentine samples with a microbrush, left to absorb for 20 s, then air-dried for 60 s [1]. The samples were subsequently placed in artificial saliva, with daily reapplication of the varnish and replacement of the saliva for seven consecutive days.
- Group 3 (Nano eggshell + Diode laser): Dentine samples in this group received the same nano eggshell application as those in group 1. Following the seven-day treatment period, the samples were thoroughly rinsed with distilled water and then subjected to diode laser irradiation [20]. This sequential protocol was performed to allow adequate interaction and deposition of the eggshell nanoparticles within the exposed dentinal tubules before laser-induced surface modification and tubule sealing. Diode laser irradiation was performed using a 940 nm diode laser (Epic X, Biolase, USA; Class IV) operated in pulse-modulated mode with 50% duty cycle at an average output power of 1.5 W for 30 s, repeated three times, resulting in a total irradiation time of 90 s [21]. Brief pauses (approximately 5 s) were allowed between successive applications to permit passive thermal relaxation of the dentine surface, and no active cooling was used.
- According to the manufacturer’s specifications, the device was operated in the pre-programmed Pain Therapy mode, in which pulse parameters are internally controlled and not manually adjustable. This mode operates at a pulse frequency of approximately 25 Hz.
- Laser energy was delivered in non-contact mode using a 300 μm fiber-optic tip at a standardized distance of 5 mm, with circular scanning motion to ensure uniform energy distribution. All procedures were performed by the same operator to ensure procedural consistency, with strict compliance with established laser safety protocols.
- Group 4 (Fluoride + Diode Laser): Fluoride varnish was applied to the dentine samples once daily for seven consecutive days, following the same protocol as that used for group 2. After the final application, samples were thoroughly rinsed with distilled water and irradiated with diode laser following the same parameters as described in group 3.
- Group 5 (Control): Samples remained untreated and were immersed in artificial saliva for seven days.

In all groups, the artificial saliva solution was replaced every 24 h to maintain consistent experimental conditions throughout the experimental period.

### SEM/EDX analysis

Dentine samples were examined by SEM/EDX (Quanta 250 FEG, field emission gun, FEI Company, Oregon, USA). Prior to examination, an approximately 25 nm thick gold layer was applied to the dentine surface via vacuum sputter coating (S150A Sputter Coater, Quorum Technologies Ltd., UK) to enhance surface conductivity and imaging resolution. SEM photomicrographs were obtained at a magnification of 3000×. Quantitative analysis of calcium and phosphorus deposits was carried out via EDX, with analytical points taken at three locations on the surface of each sample, and the resulting values of calcium and phosphorus were averaged [22].

### Assessment of the percentage of patent dentinal tubules using ImageJ analysis

SEM images were analyzed via ImageJ software (version 1.53a; National Institutes of Health, USA). The total image area was quantified automatically in µm², and the total area of patent dentinal tubules was measured. The percentage of patent dentinal tubules was determined by calculating the ratio of patent tubule area to total image area according to the following formula [23]:

$$\begin{aligned} &\text {percentage of patent dentinal tubules}=\frac{\text{Total area of patent dentinal tubules}\left({\mu\,{\text m}^2}\right)}{\text{Total image area}\left({\mu\,{\text m}^2}\right)}\times\,100 \end{aligned}$$

### Statistical analysis

Statistical analysis was conducted using SPSS (Version 27; IBM Corp., Armonk, NY, USA), GraphPad Prism, and Microsoft Excel 2016. The normality of data distribution was evaluated using Shapiro–Wilk and Kolmogorov–Smirnov tests, which confirmed normal distribution of all data. Accordingly, intergroup comparisons were conducted using one-way ANOVA followed by Tukey’s post hoc test. Statistical significance was set at *P* ≤ 0.05.

## Results

### SEM analysis

**Fig. 1:**
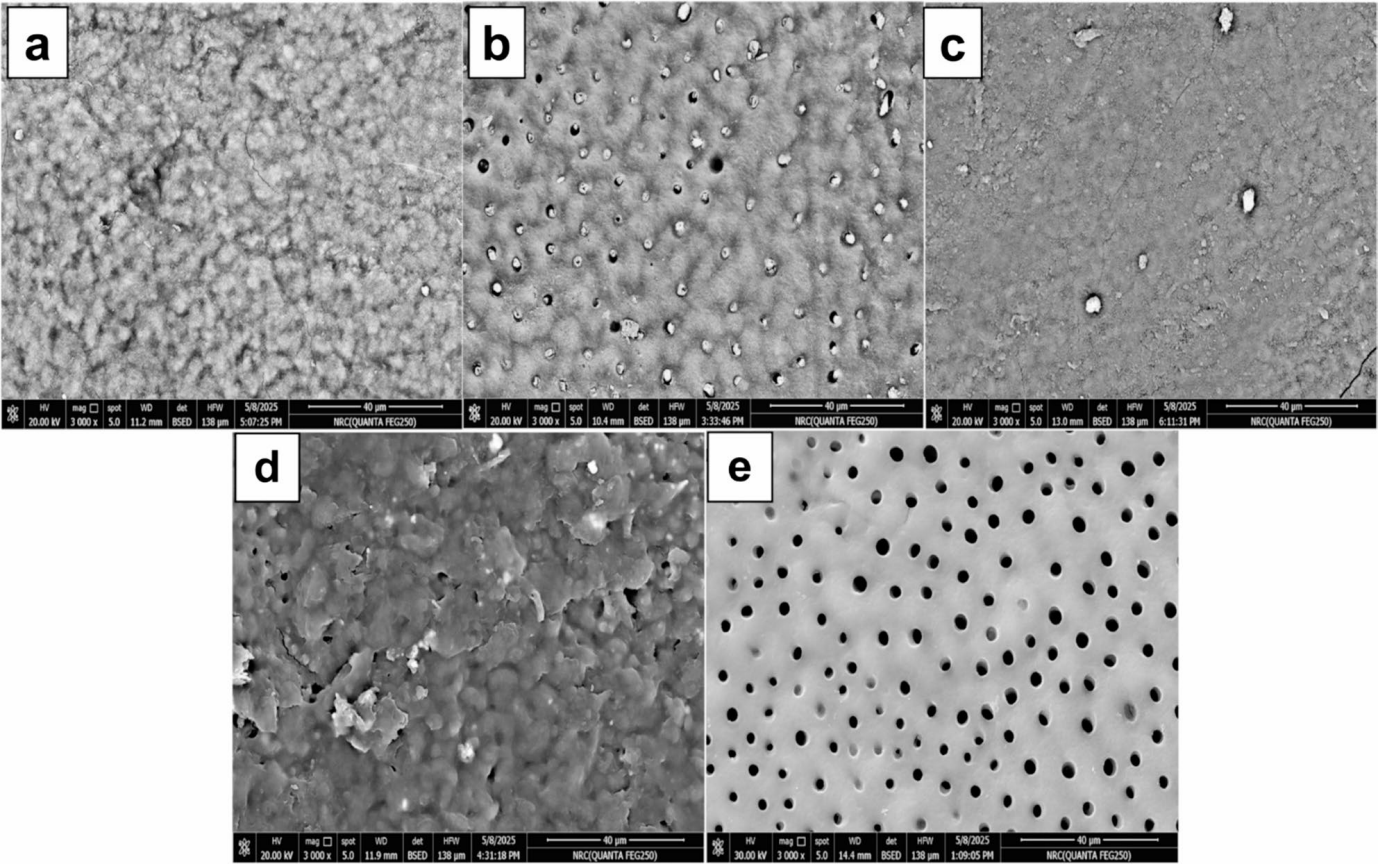
SEM photomicrographs showing dentine surface morphology of the different groups at 3000× magnification. **a** nano eggshell group showing effective tubule occlusion; **b** fluoride group exhibiting partial tubule occlusion; **c** nano eggshell + laser group showing complete tubule sealing with melted dentine surface; **d** fluoride + laser group demonstrating high degree of tubule obliteration with island-like surface appearance; **e** control group showing open dentinal tubules

The SEM photomicrographs presented in Fig. 1 show the morphological characteristics of the dentine surfaces of all groups. In the nano eggshell group, there was effective occlusion of patent dentinal tubules by mineral precipitation (Fig. 1a). Photomicrograph of fluoride group (Fig. 1b) revealed that the majority of dentinal tubules were completely occluded, with others partially occluded, and a small percentage of exposed dentinal tubules. In the nano eggshell + laser group, the dentine surface was covered by melted dentine, and the dentinal tubules were completely sealed (Fig.1c). The fluoride + laser group photomicrograph exhibited a high degree of tubule obliteration, with the dentine surface showing an island-like appearance (Fig.1d). Meanwhile, the control group photomicrograph (Fig. 1e) revealed open dentinal tubules, validating the hypersensitive dentine model used in this study.

### Percentage of patent dentinal tubules

The percentage of patent dentinal tubules for all groups is presented in Table 1. Statistical comparison was performed via one-way ANOVA followed by Tukey’s post hoc test, which revealed a statistically significant difference among the five groups (*P* < 0.0001). Group 5 (control) presented the highest mean percentage of patent dentinal tubules (12.95 ± 1.39%), followed by Group 2 (fluoride), with a mean of 6.73 ± 0.55%, and Group 4 (fluoride + laser), with a mean of 3.84 ± 0.67%. Group 1 (nano eggshell) presented a lower mean value (2.33 ± 0.21%), whereas Group 3 (nano eggshell + laser) presented the lowest percentage (0.65 ± 0.14%) of patent dentinal tubules.

**Table 1:** Mean ± standard deviation (SD) values of percentage of patent dentinal tubules of all groups:

	Minimum	Median	Maximum	Mean	SD	*P* value
Group 1 Nano eggshell	1.97	2.36	2.60	2.33 ^**a**^	0.21	< 0.0001*
Group 2 Fluoride	6.24	6.52	7.70	6.73 ^**b**^	0.55	
Group 3 Nano eggshell + laser	0.45	0.61	0.90	0.65 ^**c**^	0.14	
Group 4 Fluoride + laser	2.90	3.75	4.80	3.84 ^**d**^	0.67	
Group 5 Control	10.60	13.30	14.50	12.95 ^**e**^	1.39	

Means with different superscript letters are significantly different at *P*< 0.05

*Significant difference at*P*≤ 0.05

### Ca/P ratio

The Ca/P ratios for all groups are presented in Table 2. Intergroup comparisons were performed via one-way ANOVA followed by Tukey’s post hoc test, which revealed statistically significant differences among the five groups (*P* < 0.0001). Group 3 (nano eggshell + laser) exhibited the highest mean Ca/P ratio (1.755 ± 0.1226), which was significantly greater than all other groups (*P* < 0.05). This was followed by Group 1 (nano eggshell) (1.403 ± 0.104), Group 4 (fluoride + laser) (1.391 ± 0.3281), and Group 2 (fluoride) (1.122 ± 0.2163), with no significant difference among these three groups (*P* > 0.05). Group 5 (Control) showed the lowest mean Ca/P ratio (0.603 ± 0.09096), which was significantly lower than all other groups (*P* < 0.05).

**Table 2:** Mean ± standard deviation (SD) values of Ca/P ratios of all groups:

	Minimum	Median	Maximum	Mean	SD	*P* value
Group 1 Nano eggshell	1.267	1.408	1.532	1.403 ^**a**^	0.104	< 0.0001*
Group 2 Fluoride	0.6303	1.164	1.292	1.122 ^**a**^	0.2163	
Group 3 Nano eggshell + laser	1.57	1.77	1.9	1.755 ^**b**^	0.1226	
Group 4 Fluoride + laser	1.053	1.352	1.875	1.391 ^**a**^	0.3281	
Group 5 Control	0.502	0.6067	0.75	0.603 ^**c**^	0.09096	

Means with different superscript letters are significantly different at*P*< 0.05

*Significant difference at*P*≤ 0.05

## Discussion

Dentine hypersensitivity is a clinically challenging condition in dentistry, characterized by short, sharp pain elicited by external stimuli and typically associated with exposed dentinal tubules [24]. Previous studies have demonstrated that hypersensitive dentine exhibits a significantly greater number and larger diameter of patent tubules per unit area compared to non-sensitive normal dentine [18]. The relationship between occlusion of dentinal tubules and dentine hypersensitivity is based on the concept that reducing tubule patency may decrease fluid movement within the dentinal tubules, thereby alleviating hypersensitivity symptoms. Accordingly, one of the primary strategies for managing dentine hypersensitivity is tubule occlusion, either through forming a physical barrier or by inducing crystalline deposit precipitation within the tubules [4].

To achieve this, various therapeutic approaches have been employed, including the application of topical agents enriched in bioactive ions like calcium, phosphate, or fluoride, which induce the intratubular precipitation of insoluble compounds [25]. Additionally, laser therapy has demonstrated effectiveness by modifying dentine surface morphology, thereby creating a physical barrier against fluid movement [4].

Nanohydroxyapatite, composed of calcium and phosphate, accounts for approximately 75% of the weight of dentine and plays a crucial role in its structural integrity. It exhibits excellent biocompatibility, being both non-toxic and non-inflammatory [13]. Among the various available sources for nanohydroxyapatite synthesis, chicken eggshell was selected for the present study because of its high calcium content, biocompatibility, and cost-effectiveness. The utilization of eggshell not only reduces the cost of producing high-quality calcium sources but also supports environmental sustainability through material recycling [19]. The application of nanomaterials for dentinal occlusion represents a promising strategy for the management of dentine hypersensitivity. Due to their nanoscale dimensions, these particles exhibit a higher surface area and reactivity, facilitating deeper penetration into dentinal tubules and promoting more effective occlusion [18].

Sodium fluoride varnish (NaF) was employed in this study as a desensitizing agent. Fluoride ions enhance dentine remineralization through their strong chemical affinity for calcium ions, resulting in deposition of calcium fluoride, which promotes sealing of patent dentinal tubules. Additionally, NaF can interact with hydroxyapatite to form a more stable fluorapatite [26].

The 940 nm diode laser was selected in this study for its well documented ability to promote dentinal tubule occlusion through thermal interaction with the dentine surface. The generated heat induces melting and resolidification of dentine, leading to protein coagulation and formation of insoluble calcium complexes that effectively seal the tubules [27].

Several studies have reported that the combination of desensitizing agents with laser irradiation yields a more pronounced and sustained reduction in dentine hypersensitivity [28–30]. Desensitizing agents offer immediate pain relief by occluding exposed dentinal tubules. In addition, diode laser application not only contributes to tubule sealing but also exerts long-term therapeutic effects by modulating neural transmission and stimulating odontoblastic activity, which may lead to the formation of reparative dentine [9].

In light of these findings, this study aimed to investigate the effect of nano eggshell, fluoride varnish, and their combination with diode laser irradiation on dentinal tubule occlusion. To mimic intraoral conditions, the samples were stored in artificial saliva. It has been demonstrated that artificial saliva represents a more suitable storage medium than distilled water for in vitro investigations, serving as a reservoir of calcium and phosphorus ions, promoting their deposition within peritubular dentine and dentinal tubules and thereby reducing dentine permeability [31].

SEM/EDX was used to qualitatively assess surface morphology and quantitatively analyze elemental composition, specifically calcium and phosphorus contents. Surface morphology of dentine reflects both the extent of tubule occlusion and the structural integrity of dentine. Together, surface morphology and Ca/P ratio are considered essential indicators of treatment efficacy for tubule occlusion [18].

The findings of the current study demonstrated that all treatment groups exhibited a significantly lower percentage of patent dentinal tubules and a significantly higher Ca/P ratio when compared with the control group. Accordingly, the null hypothesis was rejected, indicating that nano eggshell and fluoride varnish, whether applied alone or combined with diode laser, effectively enhanced dentinal tubule occlusion and increased the Ca/P ratio of dentine.

Compared with the control samples, the dentine samples treated with nano eggshell presented a significantly lower percentage of patent dentinal tubules and a significantly greater Ca/P ratio. This effect could be attributed to the nanoscale hydroxyapatite particles, which enable deeper penetration into the dentinal tubules. The increased surface area resulting from the smaller particle size may have enhanced the material’s reactivity, facilitating mineral deposition within the tubules and promoting more effective occlusion [1]. These findings are supported by a previous study that demonstrated effective dentinal tubule occlusion using nanohydroxyapatite synthesized from eggshells [18]. Similarly, the findings of the present study align with those of another study, which concluded that nanohydroxyapatite gel was effective in occluding patent dentinal tubules [1].

Furthermore, the results of the present study showed that dentine samples treated with fluoride varnish exhibited a significantly lower percentage of patent dentinal tubules and a significantly higher Ca/P ratio compared to the control samples. This enhanced effect can be attributed to the prolonged fluoride retention on the tooth surface, enabled by the varnish’s viscous consistency. Upon application, it quickly dries to form a thin, transparent film that helps maintain the active agents at the site of application [30]. Consistent results have been reported with NaF varnish, which significantly enhanced the occlusion of patent dentinal tubules [32].

Although the nano eggshell group exhibited a significantly lower percentage of patent dentinal tubules than the fluoride group, no significant difference was observed in the Ca/P ratio between the two groups. This discrepancy reflects the distinct action mechanisms of the two materials. The enhanced occlusion achieved by nano eggshell results from the nanoscale particle size and increased surface area of nanohydroxyapatite, promoting deeper penetration into the dentinal tubules and enabling more uniform physical sealing [1]. In contrast, fluoride promotes calcium fluoride deposit formation, resulting in enhanced superficial remineralization but reduced occlusion efficacy owing to limited penetration within the tubules.

The absence of a statistically significant difference in the Ca/P ratios despite different occlusion effectiveness can be explained by spatial distribution differences. EDX analyzes surface composition but does not reflect the spatial distribution or depth of mineral deposition within dentinal tubules. Eggshell nanoparticles penetrate deeply, distributing minerals throughout the tubular length, whereas fluoride promotes superficial mineral deposition. Therefore, both groups demonstrated comparable Ca/P ratios, despite differences in volumetric distribution and occlusion outcomes.

Among all the groups, the nano eggshell combined with diode laser group demonstrated the best results, exhibiting the lowest percentage of patent dentinal tubules and the highest Ca/P ratio. This optimal performance may be attributed to a synergistic mechanism in which nanoscale hydroxyapatite particles penetrate deeply into dentinal tubules, facilitating mineral deposition and tubule occlusion, combined with the melting effect induced by the diode laser. Laser energy absorption by dentine minerals generates a significant increase in temperature, promoting thermochemical ablation and subsequent surface melting [13].

These findings are in agreement with a previous study [33], which reported that combining nanohydroxyapatite with laser irradiation resulted in effective dentinal tubule occlusion. This effect was attributed to the laser-induced surface melting, which facilitated the penetration of nanohydroxyapatite particles up to 10–15 μm into the dentinal tubules. Similarly, another study [13] showed that the combination of nanohydroxyapatite with laser irradiation resulted in more effective tubule occlusion compared to nanohydroxyapatite alone.

Compared with the fluoride group, the fluoride combined with diode laser group exhibited a significantly lower percentage of patent dentinal tubules. This may be due to the effect of the diode laser, which modifies the dentine surface through melting and recrystallization. Additionally, laser application may enhance fluoride ion penetration into the dentinal tubules, promoting calcium fluoride deposition and contributing to improved tubule occlusion [9]. This finding is consistent with another study [30], which concluded that fluoride varnish combined with diode laser was more effective than fluoride varnish alone, suggesting a synergistic effect between the remineralizing capability of fluoride and the thermochemical sealing properties of the laser.

Although the fluoride and diode laser group demonstrated a higher Ca/P ratio compared with the fluoride group, this difference was not statistically significant (*P* > 0.05). This finding indicates that while the combined treatment exhibited greater occlusion effectiveness, both treatment interventions yielded comparable stoichiometric ratios of calcium and phosphorus within the treated dentine matrix. The lack of a significant difference in Ca/P ratios suggests that the enhanced occlusion observed in the fluoride and laser group results from improved spatial distribution and penetration of mineral deposits rather than increased overall mineralization.

An important consideration for clinical application is whether nano eggshell particles remain stable within dentinal tubules or are prone to detachment. While this study demonstrated effective immediate tubule occlusion, long-term stability was not evaluated. Owing to their nanoscale size and calcium-rich composition, eggshell nanoparticles are capable of penetrating deeply into dentinal tubules, where they may become mechanically retained and contribute to mineral deposition within the tubules. The increased surface area associated with a smaller particle size enhances material reactivity, facilitating mineral deposition within the tubules and potentially improving integration with the dentine substrate [1], which may improve resistance to immediate dislodgement.

The application of diode laser irradiation may further enhance the stability of tubule occlusion through thermochemical surface modification, as SEM analysis of the nano eggshell + laser group revealed areas of melted dentine with completely sealed tubules. However, confirmation of long-term durability requires future studies incorporating aging protocols such as pH cycling, brushing simulation, and extended storage periods.

A key strength of this study is the application of recent technologies, such as diode laser irradiation, along with the use of nano eggshell as a novel biomaterial currently under investigation for dentinal tubule occlusion. Furthermore, the combined use of SEM and EDX allowed both qualitative and quantitative assessment of dentinal tubule occlusion, providing a comprehensive evaluation of the treatment effects.

However, this study has several limitations. As an in vitro study, while allowing controlled experimental conditions, cannot fully replicate the physiological complexity of the intraoral environment, including salivary buffering effects, dynamic pH changes, and microbial activity. Additionally, all experimental groups were assessed immediately after the 7-day treatment period without long-term follow-up; consequently, the findings cannot reflect long-term stability. Therefore, further in vivo studies with extended follow-up periods are necessary to validate the clinical relevance and durability of the evaluated treatment protocols.

## Conclusions

All tested treatment protocols significantly reduced the percentage of patent dentinal tubules and significantly increased the Ca/P ratio compared with the control group, indicating their effectiveness in managing dentine hypersensitivity. Among the evaluated interventions, the combination of nano eggshell and diode laser demonstrated the highest efficacy, suggesting a synergistic interaction between the remineralizing potential of nanohydroxyapatite and the thermochemical sealing properties of diode laser irradiation. Although fluoride (used alone or combined with laser) was effective, nano eggshell-based treatments exhibited superior performance, thus presenting a promising, biocompatible, and cost-effective treatment for dentine hypersensitivity management.

## Data Availability

The datasets used and/or analyzed during the current study are available from the corresponding author upon reasonable request.

## Abbreviations

Ca/P: Calcium to Phosphorus Ratio
SEM: Scanning Electron Microscopy
EDX: Energy Dispersive X-ray Spectroscopy
NaF: Sodium Fluoride
